# Aptamer based diagnosis of crimean-congo hemorrhagic fever from clinical specimens

**DOI:** 10.1038/s41598-021-91826-8

**Published:** 2021-06-16

**Authors:** Tahmineh Jalali, Mostafa Salehi-Vaziri, Mohammad Hassan Pouriayevali, Seyed Latif Mousavi Gargari

**Affiliations:** 1 Department of Biology, Faculty of Basic Sciences, Shahed University, Tehran, Iran; 2 Department of Arboviruses and Viral Hemorrhagic Fevers (National Reference Laboratory), Pasteur Institute of Iran, Tehran, Iran; 3 Reaserch Center for Emerging and Reemerging Infectious Diseases, Pasteur Institute of Iran, Tehran, Iran

**Keywords:** Viral infection, Assay systems

## Abstract

Crimean-Congo hemorrhagic fever (CCHF) is an acute viral zoonotic disease. The widespread geographic distribution of the disease and the increase in the incidence of the disease from new regions, placed CCHF in a list of public health emergency contexts. The rapid diagnosis, in rural and remote areas where the majority of cases occur, is essential for patient management. Aptamers are considered as a specific and sensitive tool for being used in rapid diagnostic methods. The Nucleoprotein (NP) of the CCHF virus (CCHFV) was selected as the target for the isolation of aptamers based on its abundance and conservative structure, among other viral proteins. A total of 120 aptamers were obtained through 9 rounds of SELEX (Systematic Evolution of Ligands by Exponential Enrichment) from the ssDNA aptamer library, including the random 40-nucleotide ssDNA region between primer binding sites (GCCTGTTGTGAGCCTCCTAAC(N_40_)GGGAGACAAGAATAAGCA). The K_D_ of aptamers was calculated using the SPR technique. The Apt33 with the highest affinity to NP was selected to design the aptamer-antibody ELASA test. It successfully detected CCHF NP in the concentration of 90 ng/ml in human serum. Evaluation of aptamer-antibody ELASA with clinical samples showed 100% specificity and sensitivity of the test. This simple, specific, and the sensitive assay can be used as a rapid and early diagnosis tool, as well as the use of this aptamer in point of care test near the patient. Our results suggest that the discovered aptamer can be used in various aptamer-based rapid diagnostic tests for the diagnosis of CCHF virus infection.

## Introduction

Crimean-Congo Hemorrhagic Fever (CCHF) is the most critical tick-borne viral disease of humanity caused by a negative sense RNA virus classified within the genus *Orthonairovirus* of the family *Nairoviridae*^1^. The CCHF virus (CCHFV) has a diameter of 80–100 nm with the genome consisting of three segments of RNA, designated as Small (S), Medium (M), and Large (L), according to their size. The S segment encodes the nucleoprotein (NP), the main structural protein of the virion, and serves a role in the packaging of the viral genome. The M segment encodes a precursor for the viral glycoproteins, which is converted to viral glycoproteins Gn (G1) and Gc (G2) following proteolytic cleavage^2^. The L segment encodes viral RNA polymerase, which is responsible for the synthesis of viral RNAs. Among arthropod-borne viruses (arboviruses), CCHFV has the highest genetic variation of 20%, 22%, and 31% within the S, M, and L segments respectively of different genotypes^3^.

Since 1944, when the disease was first described in the Crimean Peninsula, human cases of CCHF have been reported in more than 31 countries located in Asia, Africa, and Europe. Consequently, the disease is considered as the most geographically widespread tick-borne viral infection and the second most pervasive arboviral infection after dengue. Since 2000, the geographical spread and incidence of CCHF have significantly increased. The expansion could be rooted in the distribution of tick vectors by either international livestock trades or migratory birds^5^. Due to the high risk of outbreaks soon, the World Health Organization (WHO) has placed CCHF on the list of priority diseases for research and development for diagnosis, prevention, and control^4^. One of the areas underlined by WHO is the development of rapid diagnostic tests (RDT) that can provide a reliable diagnosis as a point of care testing^3, 5^.

In general, diagnostic methods for CCHF are divided into two groups: direct and indirect assays. Direct methods, useful in the early stage after the onset of the disease, including isolating the virus, detecting the virus antigen, and identifying the genome of the virus^6^. Indirect methods include serological techniques such as immunofluorescence, hemagglutination inhibition, complement fixation, and ELISA, for the detection of antibodies against the virus. IgM and IgG antibodies can be detected in the serum of patients, 5–7 days, and 7–9 days after the onset of illness, respectively^7, 8^.

Although antigen detection can be useful for early diagnosis, to the best of our knowledge, only one commercial kit (CCHFV-Ag-ELISA,VectorBest, Novosibirsk, Russia) is available. While molecular assays are fast, highly sensitive, and specific diagnostic methods, they are expensive and need well-equipped laboratories. This could be the main challenge in rural and remote areas where the majority of cases occur.

Considering the importance of point-of-care testing for timely diagnosis of CCHFV infection, there is a sheer necessity for the development of specific, sensitive, and easy to use RDT. Currently, there is no confirmed CCHF RDT available on the market. Recently, Coris BioConcept company has designed a CCHF IgM RDT kit; however, based on two recent studies its sensitivity is not suitable for screening purposes^9, 10^.

Aptamers are considered as an effective and powerful tool for being used in rapid diagnostic methods because of their ability to bind to a wide range of targets with high affinity, specificity and sensitivity^11, 12^.

Aptamers are single-stranded DNA (ssDNAs), RNAs, or modified nucleic acid sequences that are usually isolated during the process of SELEX's (Systematic Evolution of Ligands by Exponential Enrichment). Aptamers, as a class of ligands, have significant advantages over other diagnostic tools such as antibodies. These include temperature resistance, non-immunogenic, non-toxic, high permeability, and a high potential for chemical changes to increase stability, minimum changes in production and reproduction, cost-effective, and easy standardization^11, 13–15^.

Highly specific aptamer/aptamers can be selected toward the whole virus or one of its antigens. The NP of the CCHFV is the main protein in the structure of the virus and the most abundant viral detectable antigen in the patient's blood. The highest levels of antibodies are produced against NP^16^. Compared to other viral proteins, NP is the most conserved one among different strains of the virus, which helps to identify the disease with different virus genotypes^17^. Therefore, in this study, the NP was selected as the target for the isolation of aptamers from an aptamer library with the protein-SELEX method. Aptamers from the last round of SELEX were cloned into a pTG19-T vector (Cat No./ID: 231124, Qiagen, Hilden, Germany), sequenced, and were analyzed by Surface Plasmon Resonance (SPR) method for affinity (K_D_) determination. Finally, the Enzyme Linked Aptamer Sorbent Assay (ELASA) method was designed using aptamer as a detector and antibody against viral NP as a capture to investigate the clinical specimens.

## Results

### SELEX

**Figure 1:**
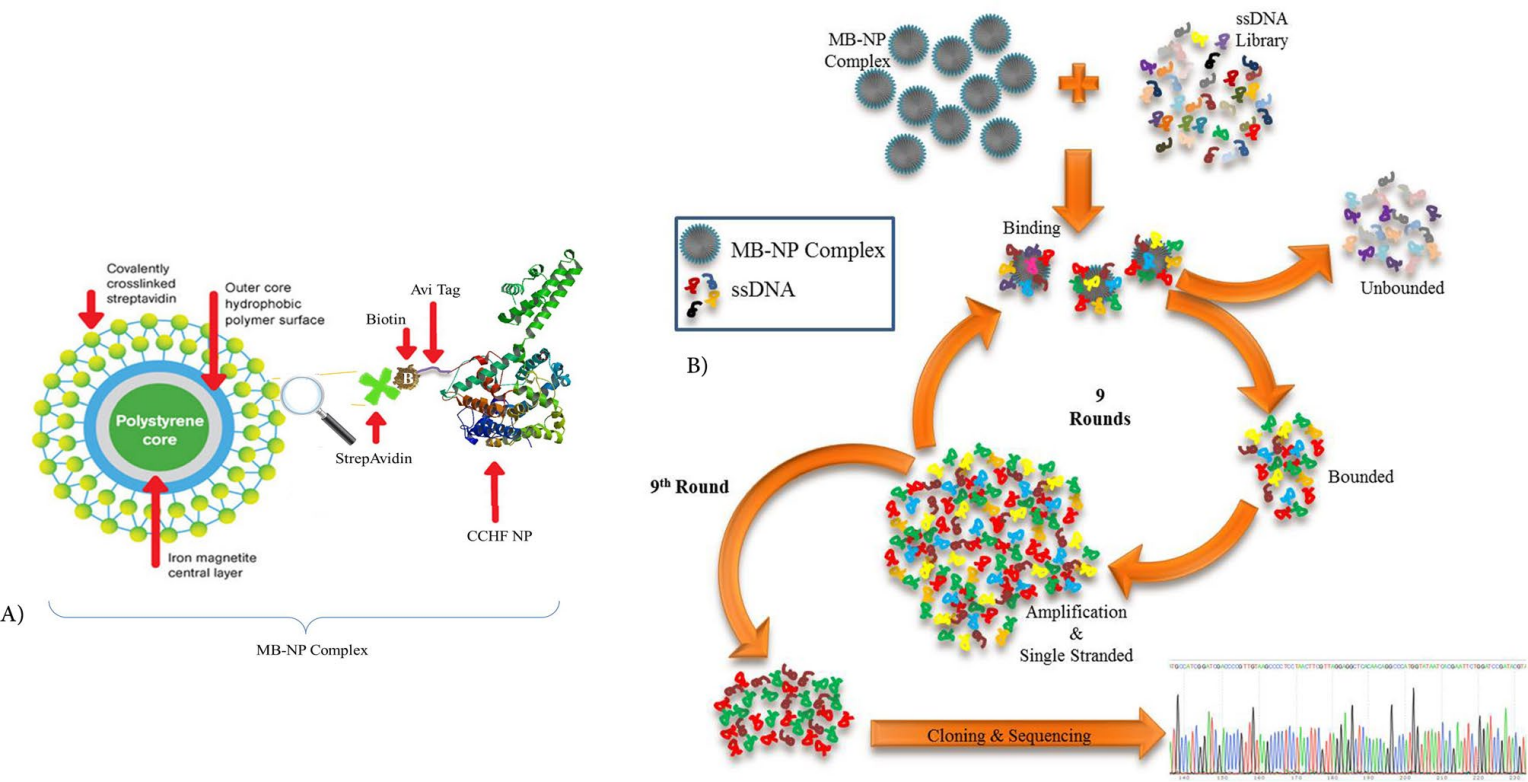
Schematic display of SELEX steps. (**A**) Schematic display of the preparation CCHFV NP-MB. The C-terminal of viral recombinant NP was biotinylated by the BirA enzyme and was fixed onto the surface of the streptavidin-coated magnetic beads. (**B**) ssDNA aptamers were mixed with Target; probable combinations of magnetic particles-NP-Aptamers (MB-NP-Apt) were deposited with a magnet. The attached aptamers were separated by heat giving after the sedimentation. The ssDNA bounded aptamers were amplified by PCR and were single-stranded by lambda exonuclease enzyme and entered the next round of SELEX. The PCR product of the optimum round was cloned and sequenced.

SELEX was performed to identify the high binding ssDNA aptamers toward CCHFV NP using an 80-nucleotide aptamer library. The recombinant NP that was fixed from the C-terminal by biotin on the surface of the magnetic bead entered to SELEX as a target molecule. The SELEX steps are schematically shown in Fig. 1.

### Choosing the optimum round

**Figure 2:**
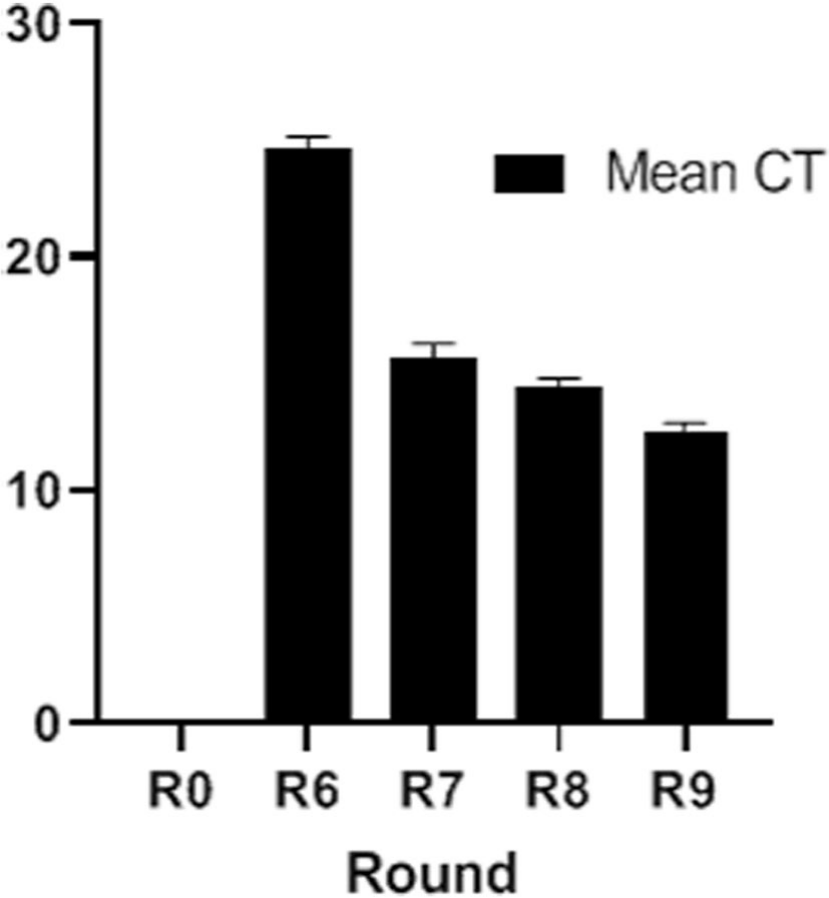
Real-Time PCR results. The result of the amplification of rounds zero (negative selection), R6, R7, R8 and R9 with the One Step SYBR TaKaRa kit. The mean values of Ct of each round have been demonstrated in the graph bar. Round 9 was selected as the optimum round with the lowest Ct value.

The same concentrations of aptamers obtained from SELEX round zero(negative selection), 6, 7, 8, and 9 were incubated with CCHFV NP for 1 h. The bound ssDNA aptamers were amplified in the same conditions with SYBR Green Real-Time RT-PCR. Cycle thresholds (Ct) of the last four rounds were compared with each other. Round zero did not show any amplification. The Mean Ct of each round were demonstrated in Fig. 2. SELEX 6 had a significantly higher Ct compared to SELEX rounds 7, 8, and 9. Therefore, based on Real-Time results, round 9 was selected as optimal round selection.

Aptamers obtained from the SELEX round 9 was cloned into a pTG19-T vector and screened with white/blue screening assay and further verified with PCR. A total of 120 positive clones were obtained and sent for sequencing. Bi direction sequences of 109 clones were assembled using CLC Mainworkbench 5.5. The sequences were verified according to the primer binding sites in both sides. The results are shown in Table 1.

**Table 1:** List of aptamers, their sequences, K_D_ values and % of occurrence.

Aptamer name	Sequences	Affinity (M)	Mean OD	Length	% of occurrence
Apta-33	CCGTAGGGTTAGGGGCGGATCGTCAGGGTGGATAAGGCA	6.62 × 10^−10^	2.73	39	9.19
Apta-1	GAAGTTAGGAGGGGCTTACAACGGGGTCAGTCCGATGGCA	5.18 × 10^−9^	2.32	40	3.67
Apta-14	TTTGATGTTAGGGGTGATGCGTGTCCTATTGACTGCACCG	9.25 × 10^−8^	0.27	40	8.28
Apta-12	GTCTTACTAGGTCAGTAAGGTACGGAGGGAACACGCGGCA	7.8 × 10^−8^	2.50	40	4.58
Apta-9	ATGCTTGTTGGTAGCGGTGGTGTTAGGGTTCGTGGGGGCA	6.85 × 10^−8^	0.21	40	2.76
Apta-8	GAAGTTAGGAGGGGCTTACAACGGGGTCGATCCGATGGCG	4.81 × 10^−8^	2.22	40	2.76
Apta-7	CGTGCCGCTTGTTCCGTAACGCCATTCGCTCGGTTGGGCA	2.71 × 10^−8^	0.19	40	3.67
Apta-22	GAAGTTAGGAGGGGCTTACAACGGGGTCGATCCGATGGCA	2.29 × 10^−8^	1.98	40	3.67
Apta-23	GAAGTTAGGAGGGGCTTACAACGGGGTTGATCCGATGGCG	1.26 × 10^−8^	2.10	40	3.67
Apta-5	GGACGATGAATTGTGATCTCATCGTTCTACTTCTGCACCG	1.09 × 10^−8^	0.23	40	5.50
Apta-15	ATAACGATACGGGGTATCGCTAGGGGTTGACTGACGTCCA	7.95 × 10^−7^	0.32	40	2.76
Apta-17	AGTTACGTCGGTTGCGAGCGTATTTGTGTTCCTGCACGC	7.5 × 10^−7^	1.88	39	0.91
Apta-2	CTAAGCAGGTAGCTATTAGCGTGCAGGGTTGGTTGTGGCA	4.03 × 10^−6^	1.56	40	2.76
Apta-3	CGATGGCAATTGCGAGGGAGTAAACCGATCGGATGGGGCA	1.08 × 10^−7^	1.79	40	6.42
Apta-4	TCTCGAAGTCCAAGGTCTAGGCTTTCGACATTGCTGCCCG	2.17 × 10^−7^	0.21	40	0.91
Apta-16	CCTTTGTCGCACCGGGATGGTTATGGGTGTTCCTCGGTCA	2.24 × 10^−7^	2.01	40	0.91
Apta-20	GAAGTTAGGAGGGGCTTACAACGGGGTCGATCCGACGGCA	2.02 × 10^−7^	2.29	40	2.76
Apta-19	CTGCGATTGAGTTGTGGGCAGTTTGCGTTCGTCCGCCGCG	1.72 × 10^−7^	0.36	40	0.91
Apta-10	CGCACGTGGTGGGGGTGAGTCCAATTAGTTGGGTTGTACA	1.38 × 10^−7^	2.09	40	0.91
Apta-13	ATCGGTCGATGTGGTTTGCGTGGAGGTGTGCAGTTGGGCA	1.2 × 10^−7^	1.78	40	0.91
Apta-21	TGGCGCCTGTGCACAGCTGGTGTGTATCTTCCGTGCTGCA	0	0.18	40	0.91
Apta-6	GAGTGCAGGAGCGGATCTAACTGCGGATACGAGTTTGGCA	0	0.18	40	2.76
Apta-18	TCGGGATGGGTTTCTTAGCGAGGGCAATTTACATGTTGCA	0	0.16	40	1.83
Apta-11	GTTGCCTCCGAGCATTATTGTGTATGTCCGTTCTGCTGCA	ND	0.98	40	0.91
Apta-24	GGCTGCGGATGGAAATAGTGGATCTCCCGTTCGTGCCGCA	ND	1.14	40	2.76
Apta-25	ATGATTGCATGGGCTGATTGTTCGGGGTGATACTTTGGCA	ND	1.90	40	2.76
Apta-26	GAAGTTAGGAGGGGCTTACAACGGGGCCGATCCGATGGCA	ND	1.78	40	0.91
Apta-27	ACTGGTCCGTAAGTGAGTTTGGGGATGGTTGGCTGCACCA	ND	1.30	40	4.58
Apta-28	AGCCCAGCAAGCTGGGGGATTATCCTGTCAGCGGAGGTCA	ND	0.93	40	3.67
Apta-29	CTCTACACATGCGTTGTCATGCATTACGTCCTTGGCAGCA	ND	0.86	40	3.67
Apta-30	GGGGGGTATCAGGTGCCGCAGGGACTATGTGCCGC	ND	1.08	35	0.91
Apta-31	ACACATAAGTGACATTGCGTGAACTCTGTCCTGCTGTGCA	ND	0.65	40	1.83
Apta-32	GAAGTTAGGAGGGGCTTACAACGGGGTCGATCCGGTGGCA	ND	1.80	40	1.83
Apta-34	CCCTTAACGACTATGCACTCCTTTCGATCGCTGTTCGGCG	ND	0.76	40	2.76

The repeated regions are underlined. The mean OD for each aptamer in the initial evaluation of aptamers in the Aptamer–Antibody sandwich was demonstrated in Mean OD column.

### Determination of K_D_ values

**Figure 3:**
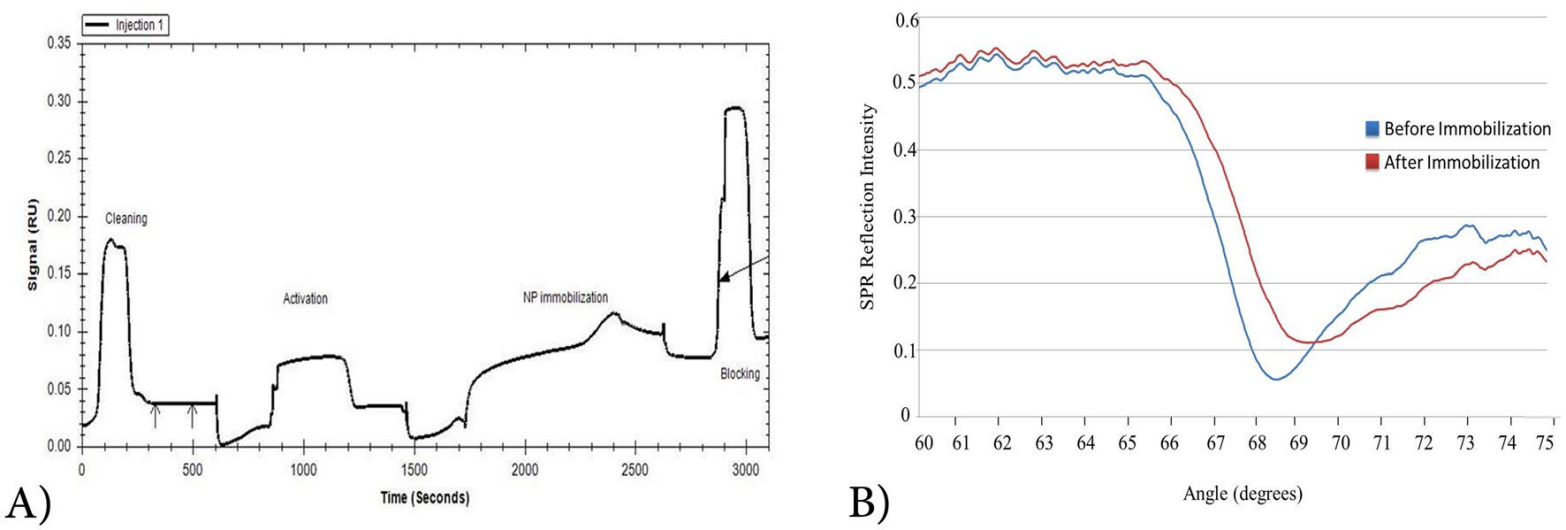
Biotin immobilization of NP on a pre-coated streptavidin chip. (**A**) Signal change during injection, (B) angle shift before and after stabilization.

**Figure 4:**
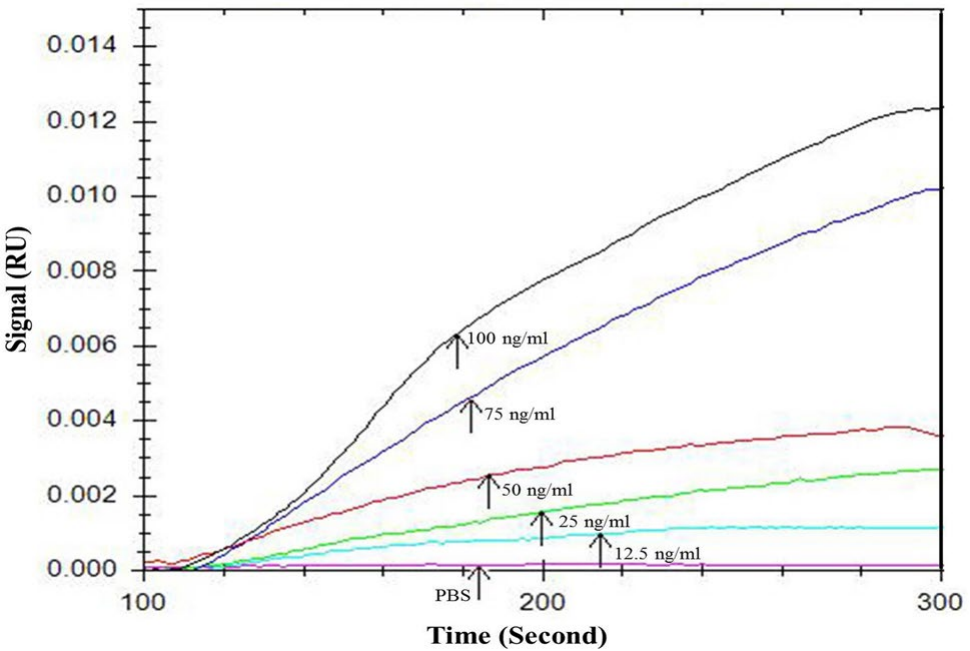
Interaction sensogram between Apt33 and CCHF-NP and calculating KD.

SPR analysis was used to determine the numerical values of K_D_. First, biotinylated recombinant NP was immobilized on the surface of the pre-streptavidin coated sensor. The signal change chart and the angel shift during the stabilization period are shown in Fig. 3. Four or five concentrations of each aptamer were used to draw binding kinetics and determine K_D_ (Fig. 4). Selected aptamers have an affinity of 4 μM–0.66 nM. The numerical values of K_D_ for the aptamers are given in (Table 1).

### Aptamer selection in the aptamer-antibody sandwich

The results of the test performed are given in Table 1 (mean OD column) as an average OD. Based on the results, aptamer 33 and a polyclonal antibody against CCHF were appropriate for the formation of the Aptamer-CCHFV NP-Ab sandwich and were used in ELASA method.

### Secondary structure prediction of aptamers

**Figure 5:**
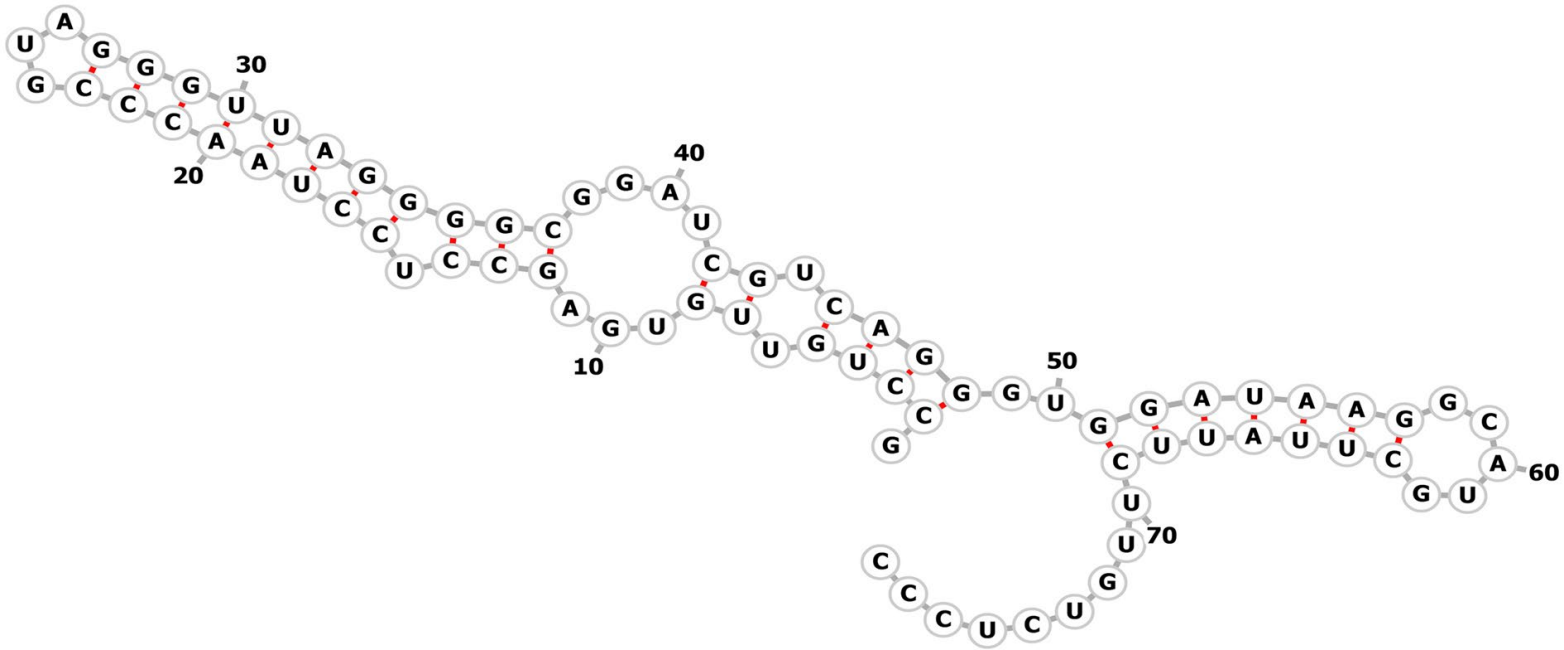
Possible 2D structures of the minimum free energy for apt33 as predicted by ViennaRNA fold.

The prediction of the secondary structure of the aptamers in this study was made with the Vienna RNA Secondary Structure Server tool. Primer regions were also considered in the study of buildings. The two-dimensional structure based on minimum free energy (MFE) was predicted and drawn with the Vienna RNA online tool (Fig. 5)^16^.

### ELASA

ELASA was performed on 77 serum samples including 49 sara from confirmed CCHF cases with Real Time RT-PCR and antigen ELISA assays, and 26 sera from patients suspected to viral hemporrhagic fevers but negative for CCHF by Real Time RT-PCR and Ag ELISA assays. Fourty eight out of 49 confirmed CCHF positive samples were also positive in our ELASA. The quantification real time RT-PCR showed positive samples in range of 5 × 10^3^–2.3 × 109 copies/µl (mean = 2.1 × 10^6^ copies/µl). The only one sample that was tested negative in the ELASA showed a laod of 2.8 × 10^5^ copies/µl. All CCHF negative specimens, and positive dengue or Chikungunya serum samples were negative (Table 2). No false positive and just one false negative results were observed, therefore, specificity and sensitivity of the test in comparison to antigen ELISA test were calculated as 100% and 97.95%, respectively.

**Table 2:**
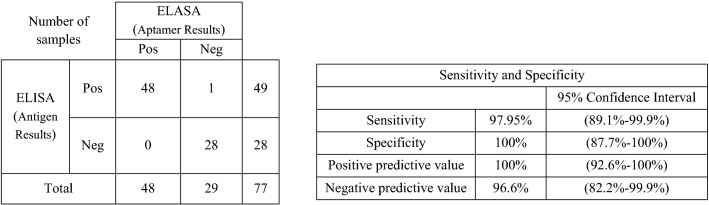
Sensitivity and specificity of the ELASA method in comparison with standard antigen ELASA test.

## Discussion

A timely diagnosis of CCHF is critical for both the management of patients and infection control. However, the definitive diagnosis of CCHF is dependent on laboratory methods because the clinical symptoms are non-specific, especially in the early stages of the disease. Despite the advantages of the molecular techniques in the diagnosis of the CCHFV, current RT-PCR techniques are unsuccessful in identifying some strains of the virus or have insufficient sensitivity^18^. Therefore, serological methods are still considered as powerful diagnostic tools. The complexity of molecular tests, their relatively high expenses, and the need for well-trained personnel, especially in remote areas with poor infrastructure facilities, are other problems concerned with these techniques. The variability of the virus genome is about 20%, whereas that of the NP is about 8%, and that is why the serological tests are preferred to the molecular method, particularly in early-stage detection and Point of Care testing^16^.

Thus there is an urgent need to develop new techniques for the efficient recognition of CCHF. Currently, available methods are based on antibodies. Antibodies have been extensively used in diagnostic procedures. Application of nucleic acid-based aptamers as diagnostic tools, along with antibodies, may increase the specificity of the test, because the smaller size of aptamer compared to antibodies (about 2–3 nm vs. 12–15 nm in diameter) allows for the binding of more recognition molecules on the same surface area of the target. The smaller size also subjects them to less steric hindrance on the surface of the target^19^.

SELEX was performed to discover ssDNA with the highest affinity towards NP of CCHFV (CCHF NP-Binding aptamer) using an 80-nucleotides aptamer library. Aptamers with a possible connection for field matrix (magnetite bead) was eliminated using negative selection at the beginning of the SELEX process. At rounds 7–9 the rate of enrichment of the isolated Aptamers was reduced and it can be assumed that the saturation of the NP level has occurred. Round 9 showed the smallest Ct, which represents the highest level of initial template^20^.

Thirty four different aptamers were selected from round 9 of SELEX procedure. SPR plot showed different K_D_ of aptamers indicating difference in their affinities to NP. The wide range of affinity from zero to 0.66 nM indicates the presence of various aptamer binding sites on the protein. NPs potentially can assemble around random RNA or ssDNA to form nucleoprotein particles^21^ similar to the native conformation in virus. Howerer, recent studies demonstrate that significant conformational changes are nessesary for RNA encapsidation^22, 23^. The role of NP in lifecyle of virus including replication, transcription and assembly are important but little data is available regarding the precise mechanism. Developed aptamers in current study had different recognition pockets on NP and may be used in future study to characterize NP-RNA interactions and even can be used as potential antiviral drugs against CCHF virus.

Selected aptamers showed affinity between 4 μM and 0.66 nM to the target. Obtaining better affinity can be due to pretreating NP with an antibody, which excludes sequences competing with the antibody for the same domain/domains and retaining those sequences binding to the pockets rather than antibody binding domains. This also fulfills the strategy of our assay system where antibody and aptamer are being used as capture and detector, respectively.

Although some aptamers including aptamers 4, 5, 7, 9, 13, 14, 15, and 19 showed high affinities toward NP, they were not suitable for use in our sandwich method assay as their ODs in ELASA were not significant. Having a common target site on the NP with antibodies could explain this finding.

After the initial screening, aptamer 33 with a 39 nucleotide in the variable region was selected for further analysis. The specificity of Apta33 was evaluated by testing whether it can bind to other arboviruses like Dengue and Chikungunya viruses. For this purpose, the Apta33 was tested by acute-phase serum samples from patients infected with Dengue virus or Chikungunya virus. Aptamer 33 did not bind and hence did not identify the proteins of these viruses while detecting CCHF-NP. The cross reactivity in this study was investigate using clinical samples suspected to other common arboviruses and viral hemorrhagic fevers in Iran (Dengue and Chikungunya viruses). As a limitation of the current study, due to lack of availability to genetically close related to CCHF virus like Hazara and Dugbe viruses, cross reactivity analysis was not performed on these viruses.

Non-specific binding of Apt33 to other human serum proteins was evaluated with serum samples from suspected CCHF patients who were negative for the disease. The negative results confirmed the lack of non-specific binding of Apta33 to other serum proteins. These studies were performed with ELASA technique where antibody and Apta33 were used as capture and detector, respectively. The sensitivity of ELASA method was about 90 ng/ml of NP in human serum.

Aptamers and antibodies have been used together to design many diagnostic methods. Our previously created ELASA method, based on antibody-aptamer for the diagnosis of *A. baumannii*, successfully detected clinical specimens with 95.47% sensitivity^24^.

The same technique was also used by Lee and Zeng, and they could detect up to 10 ng of NS1 protein from ZIKA virus. Although the method was not evaluated with clinical specimens, the potential use of selected aptamers to detect the antigen of the ZIKA virus supporting our assay system was worthwhile^20^. They further reported that aptamer-antibody sandwich assay could increase the sensitivity of ELASA test tenfold compared to the aptamer-aptamer sandwich assay^20^. Their isolated aptamers have the K_D_ values at the picomolar range while our isolated aptamers showed a K_D_ values in the nanomolar range. The higher sensitivity of their assay system compared to ours can be due to the differences in SELEX procedures such as buffering and environmental conditions of the reactions. They analyzed their isolated aptamers in the chemical buffering condition, where as we evaluated the aptamers in the human serum and physiological conditions.

Bruno^18^et al., isolated aptamers with high-specificity and affinity toward four different epitopes of CCHFV envelope, as well as formalin-inactivated CCHFV particle of strains IbAr 10200 and Drosdov (Dros)^18^. The isolated aptamers were used in ELASA method without determining the K_D_ values of the aptamers. Moreover, the sensitivity and specificity of the methods in detecting the viral antigen have not been evaluated with the clinical specimen. Bruno et al.^18^ performed their SELEX toward glycoproteins epitopes of CCHFV, which show the highest rate of variation between the different strains of the virus. In the present study, we selected the CCHFV NP as a target, which is the most conserved antigen among the seven known genotypes of the virus. This could increase the sensitivity of the test.

Phylogenetic studies so far have shown that there are seven genotypes of the virus based on S-segment^25^. Four genotypes and one outgroup circulating in Iran, including Asia-1, Asia-2, Europe-1, and Europe-2 and Out-Group Kerman-22, were used in this study^26^. The antigen from all 5 strains has been detected with ELASA and this can be considered as an important advantage of our strategy particularly in primary screening and initial diagnosis of the disease.

Bay et al. (2012) isolated Aptamer for H5N1 hemagglutinin with a K_D_ of 4.65 nM which could detect the virus in clinical swab specimens. They reported the lowest and remarkable turnaround time of 1.5-h for the Aptasensor test in comparison with the virus isolation, ELISA, PCR methods^27^. The isolated aptamer was used to design SPR biosensors. Since our strategy is mainly based on the primary diagnosis of CCHF disease in the onset of the disease as well as in outbreak investigation, therefore we preferred simple test requiring less laboratory facilities.

The importance of the secondary structure of aptamers, in binding to the target has been reported by several researchers and is used for in-sillico of design Aptamers^28, 29^. Aptamers 1, 8, 20, 22, 23, 26, and 32 showed very close 2D structures with a 26 nucleotide sequence repeat. The underlined areas in Table 1 shows repeated sequences. The results suggest that a distinct line, and possibly a specific structure, have been preferred by NP and are enriched during nine rounds of SELEXs. Interestingly Apta 33 and Apta 1, with the highest K_D_ levels, exhibited quite similar structure.

Samples used as a positive control in this study were RT-PCR and Capture Antigen ELISA (CCHFV-Ag-ELISA, VectorBest, Novosibirsk, Russia) positive samples. Based on the analyses performed in the department of Arboviruses and Viral Hemorrhagic Fevers (national reference laboratory), the ELISA kit used has a sensitivity of 97% with 100% specificity (data not shown)^30^. In our ELASA test, 90 ng of NP was detected, whereas the antibody-antibody sandwich ELISA method could detect a minimum of 170 ng of NP in serum. Using aptamers instead of antibodies as a detector could significantly improve the sensitivity of the test method.

## Conclusion

In this study, specific aptamers against CCHFV NP were isolated and presented. The efficacy of isolated aptamers was evaluated and confirmed with standard available diagnostic tests. Our ELASA assay was successfully diagnosed with clinical specimens with very high sensitivity and specificity. This simple, specific, and the sensitive method can be used as a rapid and early diagnosis tool, as well as the point of care near the patient.

## Methods

### The production of recombinant nucleoprotein (NP)

The NP was produced, as explained previously^17^. Briefly, the NP of CCHFV was designed and synthesized on pBSK ( +) simple-Amp vector and sub-cloned into the pAC4 expression vector, which adds AviTag to the 3’ end of NP. The pAC4 vector containing CCHFV-NP was transferred to *Escherichia coli* BL21 (DE3) for expression. Protein purification and simultaneous refolding were performed on the AbMCA chromatography column of Avidity, LLC (Aurora, Colorado, USA). The recombinant NP was evaluated using Circular Dichroism, Western Blotting, ELISA, and Immunofluorescence methods.

### Preparation of CCHFV NP and Magnetic Beads complex (NP-MB)

Biotin was enzymatically added to the carboxyl end of NP. The BirA enzyme (EC 6.3.4.15) activates biotin to the form of 5'-adenylate biotinyl and adds it to the AviTag at the carboxyl end of NP. The biotinylated NP was stabilized on the surface of the dynabeads magnetic beads based on the biotin and streptavidin interaction. The formation of the NP-MB complex was checked using the specific antibody for NP.

### SELEX procedure

Oligonucleotides, including the random 80-nucleotide ssDNA aptamer library (GCCTGTTGTGAGCCTCCTAAC (N_40_) GGGAGACAAGAATAAGCA) and primers, were purchased from the Metabion (GmbH, Germany ). The forward and reverse primers used were as follows: CCATGGGCCTGTTGTGAGCCTCCTAAC and GGATCCGGGAGACAAGAATAAGCA. The same primers were also used in the biotinylated and phosphorylated forms.

For the elimination of the aptamers having affinity toward MB, the first round of SELEX was performed as negative SELEX. The amount of 810 pmol of the aptamer library in PBS as a binding buffer was heated at 95° C for 5 min and was immediately cooled on an ice bath. The aptamer library was incubated at 4° C with 70 μl of MB with gentle shaking for 1 h, then were precipitated, and the supernatant which contains unbounded aptamers was removed. The supernatant was added to 70 μl of NP-MB and incubated for 90 min at 4° C with gentle shaking. The mixture was precipitated with a magnet, and the supernatant was removed. The pellet was washed with 70 μl of PBS and suspended in 75 μl of RNase DNase free water at 70° C. Fifteen μl of the suspended pellet was stored at − 20 °C, and the remaining 60 μl was entered into 4 PCR reactions as a template and amplified for the next round of SELEX.

The output of each SELEX was amplified with slight changes in PCR reaction conditions. The PCR products were single-stranded by the lambda exonuclease enzyme (Cat No: EN0561, Thermo Fisher Scientific Inc.) and were purified by ethanol precipitation. The binding conditions became gradually stringent from the 1st to the 9th rounds of SELEX by reducing the incubation time from 75 min in the first round to 15 min in the last round and increasing the washing steps from 1 to 7 times.

To assess the efficiency of SELEXs progress and terminate the SELEX procedure at the optimum round, the DNA aptamers of the SELEX rounds 0, 6, 7, 8, and 9 were analyzed by real-time PCR. The PCR product of each round was converted to ssDNA with lambda exonuclease, and the level of ssDNA was measured at 260 nm. The same concentrations from aptamers of bands zero, 6, 7, 8, and 9 were separately mixed with recombinant NP and incubated for 1 h. The supernatant containing unbounded aptamers was removed, and the pallet containing MB-NP-Apt was washed three times, and the bounded aptamers were analyzed with the real-time PCR^20^. PCR reaction was conducted in triplets, each containing 20 μl TaKaRa ExTaq SYBR kit (Cat No: RR086A, Takara Bio, Siga, Japan) and the two μl of the sample from each SELEX rounds. The analysis of the test results was performed with the Rotor-Gene Q software, and the Threshold cycle (Ct) of each step was determined.

The PCR product of last SELEX was cloned using a pTG19-T vector (Qiagen, Hilden, Germany) with the white/blue screening technology. White colonies were screened using colony PCR method and were sequenced on both direction using forward and reverse primers. The sequencing results were assembled using CLC Main Workbench 5.5 software (CLC Bio, Cambridge, MA).

### SPR evaluation of aptamer enrichment and determination of K_D_

The affinity of aptamers was measured using BioNavis Surface Plasmon Resonance (SPR) device (VASA MP-SPR NaviTM 210A VASA, BioNavis Ltd, Finland) according to the manufacturer’s instructions^31^. Briefly, PBS was used as a running buffer and pre immobilized streptavidin chip was used to stabilize biotinylated NP. After activating by running buffer (PBS, 2 M NaCl, and 0.1 M NaOH) for 2 min, the biotinylated NP at an optimum concentration of 80 μg / ml in PBS was immobilized at a surface density of 300 resonance units (RU) on the Fc1 (flow cell) of SA chips, at a flow rate of 30 mL/min for 15 min. Two different channels were used simultaneously, one as control where the only buffer was passed through, and the other channel was used for the test sample. The aptamers were injected above NP at four or five different concentrations ranged from 10 to 100 nM at a flow rate of 25 ml/min for 2 min. Kinetic parameters, including k_on_ (on-rate constant), k_off_ (off-rate constant), and apparent K_D_ (dissociation constant), were calculated using Trace DrawerTM for SPR NaviTM. The K_D_ values were calculated as the ratio of k_off_/k_on_ rate constants by using the 1:1 Langmuir interaction analyte model.

### Secondary structure prediction of aptamers

Vienna RNA Secondary Structure server of the online database and the RNA fold web server of Vienna University, Institute for Theoretical Chemistry of Vienna University at http://rna.tbi.univie.ac.at//cgi-bin/RNAWebSuite/RNAfold.cgi were used for determining the secondary structure of the Aptamers^18^.

### Selecting aptamer for the aptamer-antibody sandwich method

The selection of aptamer, with a unique binding site (rather than antibody binding site) to CCHF-NP, was important for the aptamer-antibody sandwich method used in this study. To obtain such an aptamer, the viral recombinant NP was added to the wells pre-coated with murine anti-CCHF antibody. Biotinylated aptamers was then added to the wells, and after washing with PBS, HRP labeled streptavidin (Thermofishr scientific, Cat No: SA10001) diluted 1:10000 was added to the wells and incubated at room temperature for 1 h. After washing the wells with PBS, TMB substrate was added and the reactions was stopped with H_2_SO_4_ after 15 min and the OD was measured at 450 nm. The host cell extracts containing an un-induced vector instead of CCHFV NP, and the biotinylated aptamer library, instead of high binding aptamers were used as negative controls. For each aptamer, two wells were considered, one well as a test and the other as control where BSA was used instead of the CCHFV NP.

### Aptamer-antigen ELASA of clinical samples

In the aptamer-antibody sandwich assay, a specific antibody against CCHFV NP with dilution1: 600 in PBS was coated into wells and incubating overnight at 4° C. The wells were washed three times with PBS-Tween20 (PBST) and were blocked with PBST buffer containing 3% non-fat dry milk (PBSTM). Wells was added with 100 μl of the serum and incubated at 37° C for 1 h. 100 ng of biotinylated aptamer with the highest affinity toward NP (Apt33), was added to the wells and incubated at room temperature with gentle mixing for 2 h. Conjugated streptavidin with HRP (Thermofisher Scientific, Cat No: SA10001) diluted 1:1000 was added to the wells and was incubated at room temperature with gentle mixing for 1 h. The wells were washed three times and dried after each step. The TMB substrate was added, and the reaction was stopped with H_2_SO_4_, and the OD at 450 nm was recorded.

A total of 77 human serum samples obtained from the Department of Arboviruses and Viral Hemorrhagic Fevers (National Reference Laboratory) at Pasteur Institute of Iran were tested with our ELASA. Among 77 sera samples from patients suspected to viral hemorrhagic fevers (VHFs), 49 were confirmed CCHF positive (by both Real Time PCR and antigen ELISA), along with 17 CCHF negative specimens, plus eleven positive Dengue or Chikungunya serum samples. Quantitative Real Time RT-PCR assay^32^ and Antigen ELISA (CCHFV-Ag-ELISA,VectorBest, Novosibirsk, Russia) test were used to evaluate the presence or absence of CCHFV. The quantification method of real-time RT-PCR were used to determine the copy numbers of CCHFV in clinical samples. The Real-Time RT-PCR FTD DENGUE/CHIK kit (FTD-Dengue/chik-43-64) used to confirm the presence of Dengue and Chikungunya viruses.

In our ELASA method for each sample, two wells were considered as test and control. Biotinylated Apt33 was added to the test wells and biotinylated primary library was added to the control wells.

Statistical analysis including calculation of sensitivity and specificity and confidence interval was carried out by Chi-sqare test using STATA softwar (version 9.2, Texas, USA).

## References

[1] Çevik MA, Erbay A, Bodur H, Gülderen E, Baştuğ A, Kubar A, Akıncı E (2008). Clinical and laboratory features of Crimean-Congo hemorrhagic fever: predictors of fatality. Int. J. Infect. Dis..

[2] Sanchez AJ, Vincent MJ, Nichol ST (2002). Characterization of the glycoproteins of Crimean-Congo hemorrhagic fever virus. J. Virol..

[3] Bente DA, Forrester NL, Watts DM, McAuley AJ, Whitehouse CA, Bray M (2013). Crimean-Congo hemorrhagic fever: history, epidemiology, pathogenesis, clinical syndrome and genetic diversity. Antivir. Res..

[4] Spengler JR, Bergeron É, Spiropoulou CF (2019). Crimean-Congo hemorrhagic fever and expansion from endemic regions. Curr. Opin. Virol..

[5] WHO 2018 Annual review of diseases prioritized (accessed 10.Jan.2019).

[6] Burt FJ, Swanepoel R, Shieh W-J, Smith JF (1997). Immunohistochemical and in situ localization of Crimean-Congo hemorrhagic fever (CCHF) virus in human tissues and implications for CCHF pathogenesis. Arch. Pathol. Lab. Med..

[7] Shepherd A, Swanepoel R, Leman P (1989). Antibody response in Crimean-Congo hemorrhagic fever. Rev. Infect. Dis..

[8] Raabe VN (2020). Diagnostic testing for Crimean-Congo hemorrhagic fever. J. Clin. Microbiol..

[9] Baniasadi V, Pouriayevali MH, Jalali T, Fazlalipour M, Azadmanesh K, Salehi-Vaziri M (2019). Evaluation of first rapid diagnostic kit for anti-Crimean-Congo hemorrhagic fever virus igm antibody using clinical samples from Iran. J. Virol. Methods.

[10] Papa A, Weber F, Hewson R, Weidmann M, Koksal I, Korukluoglu G, Mirazimi A (2015). Meeting report: first international conference on Crimean-Congo hemorrhagic fever. Antiv. Res..

[11] Wang T, Chen C, Larcher L, Barrero RA, Veedu RN (2018). Three decades of nucleic acid aptamer technologies: lessons learned, progress and opportunities on aptamer development. Biotechnol. Adv..

[12] Mehlhorn A, Rahimi P, Joseph Y (2018). Aptamer-based biosensors for antibiotic detection: a review. Biosensors.

[13] Kalra P, Dhiman A, Cho WC, Bruno JG, Sharma TK (2018). Simple methods and rational design for enhancing aptamer sensitivity and specificity. Front. Mol. Biosci..

[14] Azadbakht A, Abbasi AR (2019). Engineering an aptamer-based recognition sensor for electrochemical opium alkaloid biosensing. J. Mater. Sci. Mater. Electron..

[15] Ladju RB, Pascut D, Massi MN, Tiribelli C, Sukowati CH (2018). Aptamer: A potential oligonucleotide nanomedicine in the diagnosis and treatment of hepatocellular carcinoma. Oncotarget.

[16] Zivcec M, Safronetz D, Scott DP, Robertson S, Feldmann H (2018). Nucleocapsid protein-based vaccine provides protection in mice against lethal Crimean-Congo hemorrhagic fever virus challenge. PLoS Negl. Trop. Dis..

[17] Jalali T, Salehi-Vaziri M, Pouriayevali MH, Gargari SLM (2019). Evaluation of Crimean-Congo hemorrhagic fever orthonairovirus avitagged nucleoprotein for potential application in diagnosis. Iran. Biomed. J..

[18] Bruno JG, Carrillo MP, Richarte AM, Phillips T, Andrews C, Lee JS (2012). Development, screening, and analysis of DNA aptamer libraries potentially useful for diagnosis and passive immunity of arboviruses. BMC. Res. Notes.

[19] Song Y, Song J, Wei X, Huang M, Sun M, Zhu L, Lin B, Shen H, Zhu Z, Yang C (2020). Discovery of aptamers targeting receptor-binding domain of the SARS-CoV-2 spike glycoprotein. Anal. Chem..

[20] Lee KH, Zeng H (2017). Aptamer-based ELISA assay for highly specific and sensitive detection of zika NS1 protein. Anal. Chem..

[21] Hiebert E, Bancroft J, Bracker C (1968). The assembly in vitro of some small spherical viruses, hybrid viruses, and other nucleoproteins. Virology.

[22] Papageorgiou N, Spiliopoulou M, Nguyen T-HV, Vaitsopoulou A, Laban EY, Alvarez K, Margiolaki I, Canard B, Ferron F (2020). Brothers in arms: Structure, assembly and function of Arenaviridae nucleoprotein. Viruses.

[23] Kirchdoerfer RN, Abelson DM, Li S, Wood MR, Saphire EO (2015). Assembly of the Ebola virus nucleoprotein from a chaperoned VP35 complex. Cell Rep..

[24] Rasoulinejad S, Gargari SLM (2016). Aptamer-nanobody based ELASA for specific detection of Acinetobacter baumannii isolates. J. Biotechnol..

[25] Salehi-Vaziri M, Baniasadi V, Jalali T, Mirghiasi SM, Azad-Manjiri S, Zarandi R, Mohammadi T, Khakifirouz S, Fazlalipour M (2016). The first fatal case of Crimean-Congo hemorrhagic fever caused by the AP92-like strain of the Crimean-Congo hemorrhagic fever virus. Jpn. J. Infect. Dis..

[26] Chinikar S, Bouzari S, Shokrgozar MA, Mostafavi E, Jalali T, Khakifirouz S, Nowotny N, Fooks AR, Shah-Hosseini N (2016). Genetic diversity of Crimean Congo hemorrhagic fever virus strains from Iran. J. Arthropod. Borne Dis..

[27] Bai H, Wang R, Hargis B, Lu H, Li Y (2012). A SPR aptasensor for detection of avian influenza virus H5N1. Sensors.

[28] Hamada M (2018). In silico approaches to RNA aptamer design. Biochimie.

[29] Ruzzo, W. L.; Gorodkin, J., De novo discovery of structured ncRNA motifs in genomic sequences. In *RNA Sequence, Structure, and Function: Computational and Bioinformatic Methods,* pp 303–318 (Springer, 2014). 10.1007/978-1-62703-709-9_15 24639166

[30] Mertens M, Schmidt K, Ozkul A, Groschup MH (2013). The impact of Crimean-Congo hemorrhagic fever virus on public health. Antiv. Res..

[31] Douzi, B., Protein–Protein Interactions: Surface Plasmon Resonance. In *Bacterial Protein Secretion Systems, Methods Mol. Biol.*, Vol. 1615, pp 257–271 (Springer, 2017). 10.1007/978-1-4939-7033-9_21 28667619

[32] Atkinson B, Chamberlain J, Logue CH, Cook N, Bruce C, Dowall SD, Hewson R (2012). Development of a real-time RT-PCR assay for the detection of Crimean-Congo hemorrhagic fever virus. Vector-Borne Zoonotic Dis.

